# Clinicians’ Perceptions of Artificial Intelligence: Focus on Workload, Risk, Trust, Clinical Decision Making, and Clinical Integration

**DOI:** 10.3390/healthcare11162308

**Published:** 2023-08-16

**Authors:** Hamid Shamszare, Avishek Choudhury

**Affiliations:** Industrial and Management Systems Engineering, West Virginia University, Morgantown, WV 26506, USA; hs00055@mix.wvu.edu

**Keywords:** AI integration, clinical trust, decision making

## Abstract

Artificial intelligence (AI) offers the potential to revolutionize healthcare, from improving diagnoses to patient safety. However, many healthcare practitioners are hesitant to adopt AI technologies fully. To understand why, this research explored clinicians’ views on AI, especially their level of trust, their concerns about potential risks, and how they believe AI might affect their day-to-day workload. We surveyed 265 healthcare professionals from various specialties in the U.S. The survey aimed to understand their perceptions and any concerns they might have about AI in their clinical practice. We further examined how these perceptions might align with three hypothetical approaches to integrating AI into healthcare: no integration, sequential (step-by-step) integration, and parallel (side-by-side with current practices) integration. The results reveal that clinicians who view AI as a workload reducer are more inclined to trust it and are more likely to use it in clinical decision making. However, those perceiving higher risks with AI are less inclined to adopt it in decision making. While the role of clinical experience was found to be statistically insignificant in influencing trust in AI and AI-driven decision making, further research might explore other potential moderating variables, such as technical aptitude, previous exposure to AI, or the specific medical specialty of the clinician. By evaluating three hypothetical scenarios of AI integration in healthcare, our study elucidates the potential pitfalls of sequential AI integration and the comparative advantages of parallel integration. In conclusion, this study underscores the necessity of strategic AI integration into healthcare. AI should be perceived as a supportive tool rather than an intrusive entity, augmenting the clinicians’ skills and facilitating their workflow rather than disrupting it. As we move towards an increasingly digitized future in healthcare, comprehending the among AI technology, clinician perception, trust, and decision making is fundamental.

## 1. Introduction

The need for artificial intelligence (AI) in healthcare is evident. With a shortage of clinicians [1] and a growing patient population, the healthcare industry is often overworked [2], placing clinicians under a high workload. In a hospital setting, clinicians are typically required to make dynamic or real-time decisions that are interdependent and constrained by the clinical situation [3]. According to the cognitive load theory (CLT) [4], an excessive workload can negatively impact decision making. In other words, clinicians who are overwhelmed with complex tasks and time constraints, often experience a decline in their clinical decision-making efficiency [5,6]. Evidence also shows that reducing clinical workload can positively influence decision-making quality in clinicians and improve patient outcomes [7,8].

AI technologies can significantly assist clinicians with their clinical workload. It can allow them to have more in-person time with their patients and potentially speed up the treatment process and augment clinical decision making. AI in healthcare aims to harness the power of advanced computational techniques and algorithms to analyze and interpret extensive and complex medical datasets, consequently aiding clinical decision making [9,10,11,12,13]. Numerous studies have demonstrated the potential of AI to augment clinical procedures and patient safety [9,10,11]. However, the benefits of AI can be realized when the end user, that is, the clinician, can use it effectively (correctly) and efficiently (timely) [14]. 

Despite all the promising evidence of AI, why does the healthcare industry not widely adopt the technology [15,16,17,18]? Unfortunately, AI in healthcare is often perceived as a complex and hard-to-use technology that requires extensive training and additional education [19,20]. If AI integration into the clinical workflow requires clinicians to perform additional tasks, it is likely that they will not adopt this assistive technology [21]. Studies have acknowledged that the lack of clinicians’ involvement in AI development, low trust in the technology, the limited explicability of AI algorithms, and unclear policy around AI accountability are factors hindering its adoption [15,16,17,18,22]. Others have identified factors such as the perceived risk of AI, expectancy, past experiences with AI, and AI knowledge as factors steering AI adoption in healthcare [16,19,23,24,25,26,27,28,29]. Therefore, it is important to understand how clinicians perceive AI.

User trust in AI has been one of the most important factors discussed in the literature [22]. Research has established a significant relationship between trust and workload [30,31,32]. For example, a study based on the Markov decision process developed a dynamic workload–trust model to assess workload based on the variation of human trust in the automated systems [33]; the study suggested that automation that lowers the workload imposed on humans gains more user trust [33]. Another study proposed a framework for quantitative and qualitative analysis of the interactions between clinicians and AI in healthcare management, considering the potential effects of workload on clinicians’ trust in AI [22]. Therefore, improving user trust in AI can potentially improve its adoption and use [27,29,34].

Research also indicates that user trust positively correlates with decision making [35,36,37,38]. For example, a study that assessed the impact of multidimensional trust on consumers’ adoption decisions in mobile financial services reported a positive association between trust and decision making [39]. Another study identified trust in technology as a significant factor that positively impacts human decision making when delegating tasks to robots [40]. A study investigated factors influencing people’s perceptions of trust in different decision-making scenarios and concluded a positive correlation between trust and AI-infused decision-making processes [38]. Therefore, user trust in AI can increase AI-driven decision making. Furthermore, prospect theory [41] suggests that individuals make decisions based on their perceived risk [42] and in the context of healthcare, the likelihood of making Ai-driven decision can depend on the perceived risk of using the technology.

**Figure 1:**
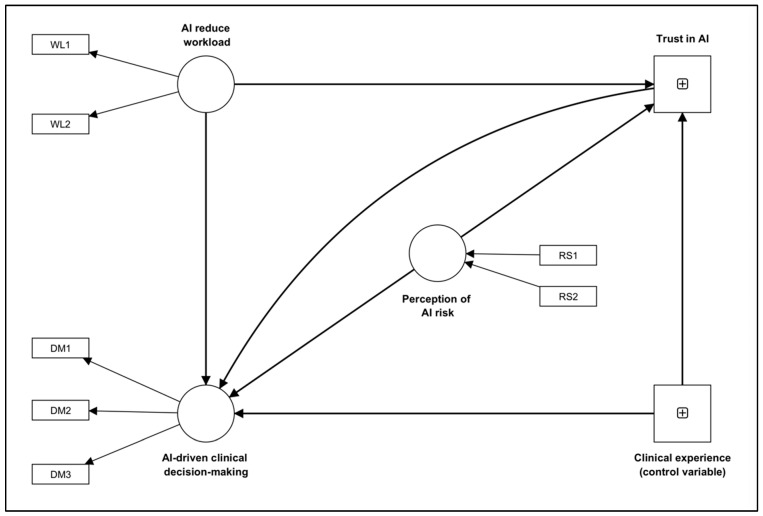
The proposed exploratory conceptual model illustrating the relationships among AI workload (WL), trust, AI-driven clinical decision making (DM), AI risk (RS), and clinical experience. In the framework, DM (1:3), RS (1:2), and WL (1:2) represent questions as indicators of these constructs.

This study explores how healthcare practitioners in the United States perceive healthcare AI, focusing on their perception of AI-induced workload, AI risk, trust in AI, and AI-based clinical decision making. As illustrated in Figure 1, we explore the following hypotheses.

**Hypothesis** **1** **(H1).** *The perception of AI’s effect on clinical workload will determine clinicians’ trust in the technology. In other words, if clinicians perceive AI as a technology that can reduce their workload, their trust in it will increase*.

**Hypothesis** **2** **(H2).** *Clinicians with more trust in AI will perceive it as a technology that can help them with clinical decision making*.

**Hypothesis** **3** **(H3).** *An increase in the perception of risks associated with using AI in clinical tasks will negatively correlate with clinicians’ likelihood of decisions based on AI-driven clinical recommendations*.

**Hypothesis** **4** **(H4).** *The perception of AI’s effect on clinical workload will determine how clinicians perceive AI-driven decision making. In other words, if clinicians perceive AI as a technology that can reduce their workload, their perception of AI-driven clinical decision making will be positive*.

Additionally, to provide a tangible application of our study’s findings, we discuss three hypothetical scenarios of AI integration in healthcare settings. Further research is needed for confirmation. These scenarios—no AI integration (Scenario A), sequential AI integration (Scenario B), and parallel AI integration (Scenario C)—each signify distinct approaches to implementing AI within the clinical workflow, illustrating how the perceptions and experiences of clinicians that we identified through our survey may manifest in real-world clinical settings (see Discussion section). Through these scenarios, the study emphasizes the importance of thoughtfully strategizing AI integration in healthcare settings to capitalize on the potential benefits while minimizing perceived risks and potential disruption to existing workflows.

## 2. Methods and Materials

The study obtained ethical approval from the Stevens Institute of Technology, Hoboken, NJ, USA (IRB ID 2022–007). We distributed an online semi-structured survey to the active healthcare practitioners residing in the United States. We collected the data from February 2021 to July 2021. 

### 2.1. Survey Items and Variables

Table 1 demonstrates the descriptive statistics of the survey questions utilized in this study. Based on the question, we developed three latent constructs: Decision Making, Workload, and AI Risk. The survey also had additional questions to capture the perceived trustworthiness of AI (single-item question). The questions were adapted from validated and well-established scales: the modified NASA-TLX [43] and the extended unified theory of acceptance and use of technology (UTAUT-2) [44] models. Participant responses to all the questions were captured using a seven-point Likert scale ranging from “strongly disagree “ to “strongly agree.” However, the scaling was inverted for certain items, with “strongly agree” being the lowest value and “strongly disagree” being the highest. To ensure consistency across all variables, these items were reverse-coded to align with the “strongly disagree” to “strongly agree” scale (see Table 1). Additionally, to evaluate the participants’ experience duration, a five-point Likert scale question was utilized, offering choices ranging from 0 to 5 years as the lowest option and 11 to 15 years as the highest option. As indicated in Table 1, we reverse-coded some questions such that a higher response value indicates a higher value in the corresponding construct. 

The survey instrument also included questions to measure the respondents’ demographics, familiarity with AI, clinical experience, and past AI experience (see Table 2).

### 2.2. Statistical Analysis

First, we tested for all the constructs’ convergent and discriminant validity. To determine how well the model explains the target constructs of interest, the convergent and reliability were assessed using four criteria [45]: factor loadings (greater than 0.50), variance inflation factor (VIF) (less than 5), composite reliability (CR) (greater than 0.70), and average variance extracted (AVE) (greater than 0.50). The factor loading represents the strength of association between each item and its corresponding construct. The VIF assesses the collinearity among the latent variables (constructs). The AVE indicates the proportion of variance in the items that can be attributed to the construct. The CR represents the internal consistency of the constructs.

After validating the latent construct (measurement model), we leveraged partial least squares–structural equation modeling (PLS-SEM) to assess the proposed hypotheses. The PLS-SEM method is a well-established method for multivariate analysis. It allows for estimating complex models with several constructs, indicator variables, and structural paths without imposing distributional assumptions on the data [46]. PLS-SEM is also suitable for small sample sizes when models comprise many constructs and items [47]. Thus, PLS-SEM is a good method for exploratory research as it offers the flexibility needed for the interplay between theory and data [48]. The structural model fit was determined using R-squared, where values of 0.75, 0.50, and 0.25 are considered substantial, moderate, and weak [46].

## 3. Results

### 3.1. Respondents

Two hundred sixty-five complete responses were retained for the analysis. About 84% identified themselves as females, and about 77% as White Americans. The most common clinical expertise areas among the respondents were family medicine (17%), geriatrics (17%), and pediatrics (11%). A total of 35% were registered nurses, 11% were nurse practitioners, 8% medical doctors, 6% residents, and the remainder were others (occupational therapists, pharmacists, medical technologists, dentists, and psychiatrists). Most respondents reported having between 0 to 5 years of clinical experience (36%), followed by 6 to 10 years (22%) and 11 to 15 years (15%). Nearly 45 (17%) respondents reported using AI in their practice. Among those who had used AI, 31% found it challenging to learn, and 38% believed it required a strong understanding to use effectively. Some found it valuable and easy to use. Most surveyed practitioners wanted AI to assist with taking clinical notes and identifying high-risk patients. They also suggested that governing bodies should establish protocols for AI use in healthcare and for shared responsibility between practitioners and AI systems. Many perceived AI as expensive and had concerns about its effectiveness in a clinical setting, lack of necessary protocols, accountability, the “black box” effect, and potential patient harm.

### 3.2. Measurement Model

Table 3 presents the factor loading, variance inflation factor (VIF), average variance extracted (AVE), and composite reliability (CR) values for the reflective constructs Workload (WL) and Decision Making (DM). Table 2 presents the factor loading values, indicating that each set of items contributed significantly to measuring its corresponding latent factor. The AVE values demonstrate that all constructs possessed convergent validity. The VIF values show minimal multicollinearity among the latent variables. All model constructs’ CR values were greater than 0.7 [49].

### 3.3. Structural Model

Table 4 presents the results of a partial least squares–structural equation model (PLS-SEM) analysis, which was used to test four hypotheses related to the relationship between clinicians’ perceptions of AI’s effects on clinical workload, trust in AI, perceptions of AI risk, and AI-driven clinical decision making. 

The analysis indicates a statistically significant and positive relationship between the perceived reduced workload due to (a) AI and trust in AI (path coefficient of 0.660, *p* < 0.001) and (b) AI-driven clinical decision making (path coefficient of 0.739, *p* < 0.001). This suggests that when healthcare professionals perceive AI as a technology that can reduce their workload, they are more likely to trust it and engage in AI-driven clinical decision making. Therefore, we fail to reject H1 and H4.

Additionally, the results indicate a positive relationship between trust in AI and AI-driven clinical decision making (path coefficient of 0.109, *p* = 0.210). However, the effect was not statistically significant. Therefore, we reject H2. This suggests that healthcare professionals with more trust in AI may not necessarily perceive it as a technology that can aid clinical decision making.

The results also show a statistically significant negative relationship between the perception of AI risk and AI-driven clinical decision making (path coefficient of −0.347, *p* < 0.001). This suggests that healthcare professionals who perceive greater risk associated with AI are less likely to engage in AI-driven clinical decision making. The relationship between AI risk and trust was not significant. Therefore, we fail to reject H3.

**Figure 2:**
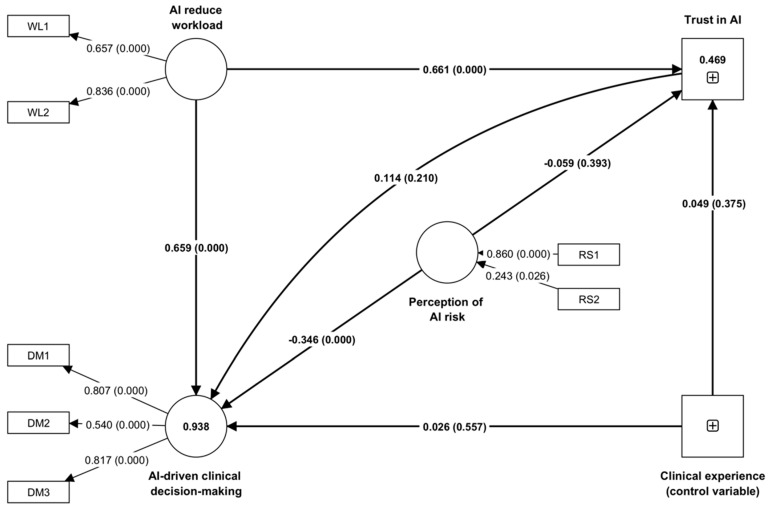
Schematic illustration of the findings from structural equation modeling showing the standardized coefficients, significance (*p*-value), and R-squared values, where DM, RS, and WL represent questions as indicators of constructs. Specifically, DM represents questions as indicators of AI-driven clinical decision making, RS represents questions as indicators of perception of AI risk, and WL represents questions as indicators of AI-reduce workload construct.

The table also shows the effect of the control variable of “clinical experience”, which was used to adjust for the potential confounding effect on the relationship between the independent variables (perception of AI risk, reducing workload) and the dependent variables (AI-driven clinical decision making, trust in AI). The results show that the effect of clinical experience was not statistically significant on either outcome variable and did not correlate with the relationship between the independent and dependent variables. The path coefficients are also illustrated in Figure 2, which shows a schematic representation of the findings from the structural equation modeling. This figure visually represents the direction and strength of the relationships among the constructs as identified in the hypothesis testing.

## 4. Discussion

This study identifies factors correlating with clinicians’ trust in AI and perception of AI-driven clinical decision making. According to this study, the perception of AI-reduced workload and AI-driven clinical decision making positively correlates with trust in AI. In contrast, the perception of risk does not significantly affect trust in AI. Moreover, the perception of AI-reduced workload correlates with AI-driven clinical decision making positively, while the perception of risk correlates with AI-driven clinical decision making negatively. The results of the PLS-SEM analysis, including the control variables of clinical experience, suggest that clinical experience, as a control variable, does not impact clinicians’ trust in AI or the AI-driven decision-making process. This finding aligns with prior research on blockchain adoption, which found a lack of correlation between years of work experience and trust and decision making [50].

### 4.1. Trust in AI

Our analysis revealed a negative relationship between trust and workload, consistent with prior research [33,51,52,53]. The results align with the social exchange theory [54], which posits that individuals develop a sense of obligation to reciprocate positive treatment from their social exchange partners (e.g., the organization). Trust is a crucial factor in developing and maintaining social exchange relationships [55]. Following the social exchange theory, an empirical analysis of a telecommunication company survey suggested that workload reduction and sharing are positively related to interpersonal trust in organizations [53].

Our study found no significant association between risk and trust, failing to support the risk management theory [56]. According to this theory, when individuals perceive high levels of risk, they may become more cautious and less likely to trust others. However, the relationship between trust and risk seems to differ in human–machine or human–technology interactions. For example, a study in the context of autonomous vehicles stated that at a high level of perceived risk, detailed explanations about the technology and no explanations led to the lowest and highest values in trust, respectively. However, these effects were reversed at low levels of the perceived risk [57]. Another study observed that during the initial interaction with automation systems, drivers’ perceived risk was primarily based on their presumptions (expectations), which may alter after using the car. The participants in the study reported the highest level of trust, perceived automation reliability, and the lowest level of perceived risk when presented with information about a highly reliable system and when driving in a low-risk situation [58]. 

The difference between our findings and the results in the literature regarding the relationship between trust and risk could be explained based on situational variations and the dynamic nature of trust. To elaborate more, trust and risk may not be correlated in certain situations, such as when the perceived level of risk is very high or very low [57]; trust is a dynamic construct that can change over time. An individual may have a high level of trust in an entity at one point and a low level in another [58]. This may make it hard to correlate trust with risk. Further research is required to confirm this relationship.

### 4.2. Decision–Making Using AI

Our findings show that the perception of AI workload positively relates to AI-driven clinical decision making, thereby supporting the limited capacity model of motivated mediated processing theory. Based on this theory [59], individuals have limited cognitive resources or attention that can be allocated to decision-making processes [60]. When cognitive resources are depleted, individuals are more likely to use mental shortcuts or simplified rules of thumb in making decisions, increasing the likelihood of errors [61]. Several other studies have also supported the idea that workload and decision making are related; for example, using an electronic clinical decision-support tool to enhance medical decision making leads to decreased cognitive workload in a simulated setting [62]. Another study assessing the decision-making processes of examiners in an observation-based clinical examination reported that cognitive processes in complex situations could be correlated with mental workload. The study suggested that an increased workload can hinder decision-making abilities [63].

Our findings support the prospect theory and identify a negative relationship between risk perception and AI-driven decision making. Prospect theory [64] explains how risk affects decision making. It argues that, for decision making, people are more sensitive to losses than to gains, a phenomenon known as “risk-seeking for gains, risk-aversion for losses.”

### 4.3. Recommendation for Better AI Integration to Support AI-Driven Decision–Making

In this study, we have examined the relationship between healthcare professionals’ trust in AI, their perception of AI risk and workload, and the impact of AI on clinical decision making. As we discuss these findings, we propose optimal integration approaches for AI in clinical workflows, which we believe could enhance clinicians’ trust in AI, positively alter their perceptions of AI risk and workload, and improve their perception of AI-aided clinical decision making.

Let us consider three hypothetical scenarios that involve a patient visiting a clinic for a pneumonia diagnosis using an X-ray image. In Scenario A, diagnosis occurs traditionally without AI involvement. Scenarios B and C propose different methods for integrating AI into clinical workflows. By juxtaposing these scenarios against our survey findings, we gain valuable insights into how AI’s practical integration into clinical workflows might influence clinicians’ perceptions of AI risk, trust in AI, and the consequential effect on their clinical decision making.

**Figure 3:**
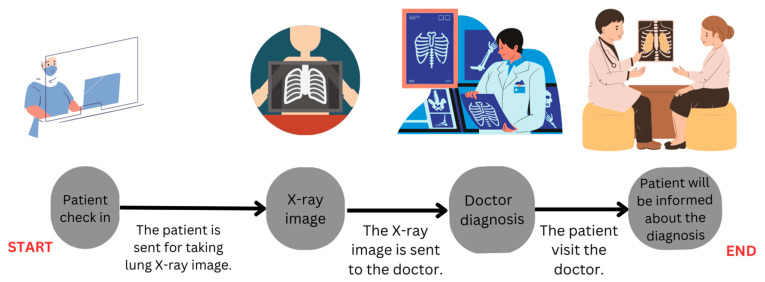
Scenario A: The first scenario for diagnosing pneumonia with no AI-based assistance.

In Scenario A (Figure 3), the clinician accesses the X-ray image and delivers the diagnosis to the patient. Here, the quality of care, particularly the diagnosis, heavily depends on the clinician’s expertise. This scenario typically entails minimal risk; however, as the clinician’s workload increases, the possibility of errors due to fatigue, burnout, or limited cognitive resources also heightens. This risk could be further magnified in low-resource clinics or when attending critically ill patients.

**Figure 4:**
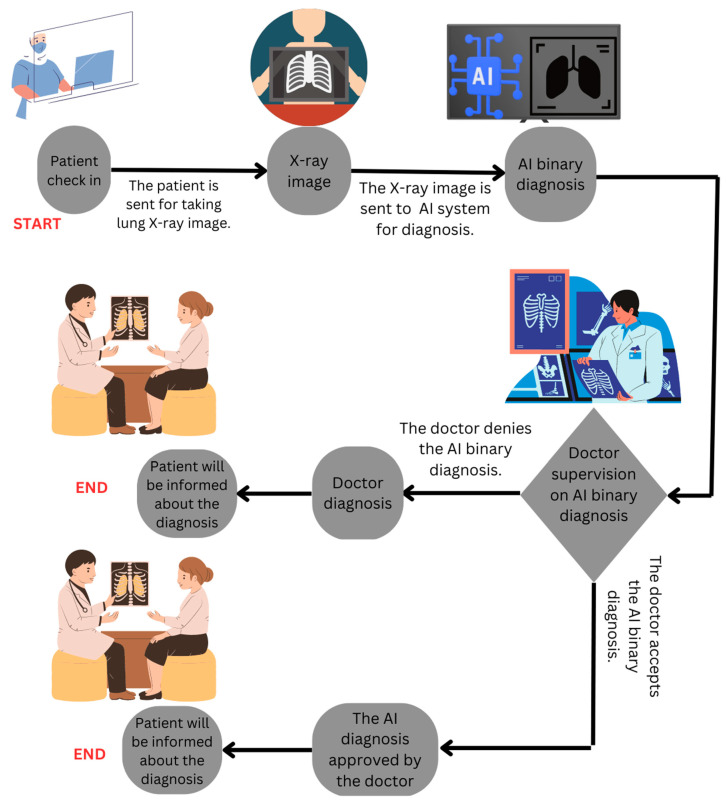
Scenario B: The second scenario for sequential diagnosis of pneumonia with AI-based and doctor.

We introduce an AI system in Scenario B (Figure 4) to alleviate this workload and associated risks. The integration of AI in this scenario is sequential (Patient → AI → Clinician → Patient). The AI system makes a diagnosis and sends it to the doctor for approval. In this stage, the doctor accepts or refutes the AI diagnosis. If the doctor accepts and approves the AI diagnosis, the diagnosis gets delivered to the patient. If the doctor rejects the AI diagnosis, it gets overridden, and the doctor communicates the final diagnosis based on their judgment. In such sequential AI integration, a doctor is required to approve or reject the AI diagnosis which could disrupt their workflow and potentially lead to added workload and underutilization of the AI system (aligns with H4).

**Figure 5:**
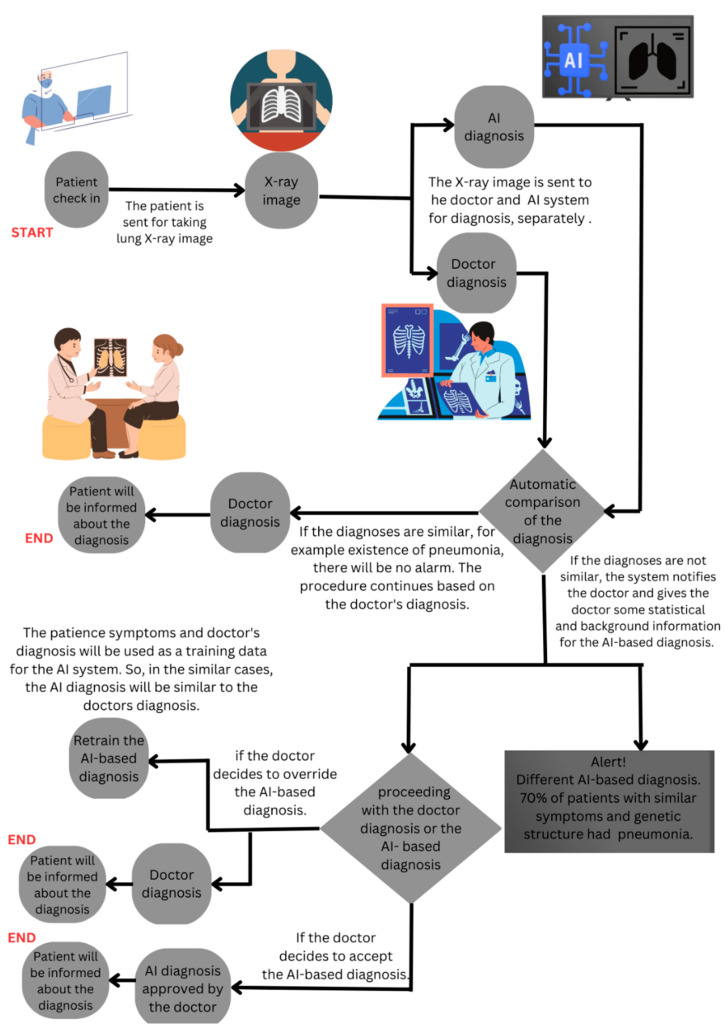
Scenario C: The third scenario for diagnosing pneumonia with AI-based assistance parallels the doctor’s diagnosis.

In contrast, Scenario C (Figure 5) posits a model where the AI system runs parallel to the clinical workflow. The AI and the clinician independently generate their diagnoses, and only in case of a discrepancy does the AI system alert the clinician. The parallel integration allows the doctor to retrain AI by rejecting its recommendation or reconsider their initial judgment without added workload of AI verification. In the third scenario, AI’s capabilities can be harnessed as a powerful tool to augment and support doctors to mitigate the risks inherent in clinical decision-making. Our survey findings demonstrate that the perception of AI reducing workload correlates with trust in AI and the perceived impact on clinical decision making. Scenario C aligns with these findings, wherein AI operates as a supportive tool, providing an additional analysis layer without unnecessary interruptions, potentially reducing the perceived workload and fostering trust. Furthermore, clinicians’ trust in AI showed a positive, albeit non-significant, association with AI-driven clinical decision making. This pattern is also likely in Scenario C, wherein clinicians can build trust in AI by understanding and correcting the AI’s reasoning, thus enhancing their willingness to incorporate AI into their decision-making process.

Overall, these scenarios illuminate the potential benefits of a parallel integration of AI into clinical workflows (Scenario C) over a sequential one (Scenario B), with potential positive impacts on clinicians’ perceptions of AI risk, trust in AI, and their willingness to adopt AI in clinical decision making. Note that these hypothetical scenarios require further research for confirmation. Caution should be exercised when generalizing these results.

### 4.4. Limitations

This study has some limitations that should be acknowledged. Firstly, this study did not find a significant impact of clinical experience as a control variable on clinicians’ trust in AI or the AI-driven decision-making process. This finding contradicts existing evidence suggesting that clinical expertise can influence trust in AI. The specific context in which AI was utilized and the limited scope of participants’ exposure to AI technologies may have contributed to this non-significant relationship. Caution should be exercised when generalizing these results, as they may not fully capture the nuanced relationship between clinical experience and trust in AI.

Further research with larger and more diverse samples is needed to better understand the influence of clinical experience on trust in AI within healthcare settings. Secondly, the study was conducted based on a cross-sectional survey. Future studies should use longitudinal data and examine the proposed relationships over time. Finally, another limitation of our study is that only a small proportion of participants (17%) reported using AI in their practice. It is important to acknowledge that the low percentage may not necessarily reflect the actual usage of AI among all participants. Many participants may be utilizing AI in their practice without being aware of it and vice versa. This lack of awareness could be attributed to various factors, such as a lack of understanding about the specific applications of AI or the absence of clear recognition of AI technologies within their practice settings. Therefore, the reported usage rate might not provide a comprehensive picture of the actual integration of AI in the participants’ professional activities. Future studies could explore participants’ levels of awareness and knowledge regarding AI to better understand its utilization in their practice.

## 5. Conclusions

Our study demonstrates the critical role of artificial intelligence (AI) in healthcare, especially in improving clinical decision making and reducing clinician workload. Our findings reveal a significant positive relationship between the perceived reduced workload due to AI and trust in AI and the adoption of AI-driven clinical decision making. Moreover, our results highlight that the perception of AI-related risks can negatively impact trust in AI and the inclination towards AI-driven clinical decision making. While the direct role of clinical experience was found to be statistically insignificant in influencing trust in AI and AI-driven decision making, further research might explore other potential moderating variables, such as technical aptitude, previous exposure to AI, or the specific medical specialty of the clinician.

By evaluating three hypothetical scenarios of AI integration in healthcare, our study elucidates the potential pitfalls of sequential AI integration and the comparative advantages of parallel integration. In conclusion, this study underscores the necessity of strategic AI integration into healthcare. AI should be perceived as a supportive tool rather than an intrusive entity, augmenting the clinicians’ skills and facilitating their workflow rather than disrupting it. As we move towards an increasingly digitized future in healthcare, comprehending the dynamics among AI technology, clinician perception, trust, and decision making is fundamental. 

## Figures and Tables

**Table 1:** Descriptive statistics of study variables (n = 265).

Survey Items	Likert Scale	Standard Deviation
I think using AI would improve my clinical decision-making skills/abilities. **(DM1)**	7	1.448
^+^ I think using AI would confuse me and hinder my clinical decision-making skills. **(DM2)**	7	1.399
I think using AI would allow me to accomplish clinical tasks more quickly. **(DM3)**	7	1.378
I think AI in healthcare is trustworthy. **(TR)**	7	1.354
I think using AI for my clinical work will put my patients (health) at risk. **(RS1)**	7	1.350
I think using AI will put my patients’ privacy at risk. **(RS2)**	7	1.549
Overall, I think using AI to complete clinical tasks will be: (very demanding—very easy). **(WL1)**	7	1.323
I think using AI in my clinical practice will reduce my overall workload. **(WL2)**	7	1.483
For approximately how many years have you been serving in your current position?	5	1.462

^+^ Reverse-coded.

**Table 2:** Participant characteristics.

	Survey Items
1	With which gender do you identify yourself with?
2	With which race do you identify yourself with?
3	What is your clinical expertise?
4	What is your designation?
5	For approximately how many years have you been serving in your current position?
6	Have you ever used any AI in your work or research?
7	How was your overall experience of using AI?
8	Given a chance, how do you want AI to assist you in clinical tasks?
9	What can the government do to motivate you to adopt AI in your clinical practice?
10	What are the factors preventing you from using AI?

**Table 3:** Collinearity statistics. Convergent validity and reliability measures of all the latent constructs.

Constructs	Items	Factor Loading	Variance Inflation Factor	Cronbach’s Alpha	Composite Reliability	Average Variance Explained
Perception of AI risk (RS) *	RS1	0.98	1.30	na	na	na
	RS2	0.65	1.30			
AI reduces workload (WL)	WL1	0.66	1.43	0.71	0.74	0.57
	WL2	0.84	1.43			
AI-driven decision making (DM)	DM1	0.82	2.15	0.75	0.81	0.54
	DM2	0.51	1.24			
	DM3	0.83	2.03			

Note: na: not applicable. * formative construct.

**Table 4:** Direct, indirect, and total effects.

Conceptualized Paths	Standardized Path Coefficient	Standard Deviation	T Statistics	*p* Values
**Direct effects**				
AI reduces workload → AI-driven clinical decision making	0.659	0.108	6.089	<0.001
AI reduce workload → Trust in AI	0.661	0.080	8.252	<0.001
Clinical experience (control variable) → AI-driven clinical decision making	0.026	0.045	0.588	0.557
Clinical experience (control variable) → Trust in AI	0.049	0.056	0.888	0.375
Perception of AI risk → AI-driven clinical decision making	−0.346	0.063	5.477	<0.001
Perception of AI risk → Trust in AI	−0.062	0.070	0.854	0.393
Trust in AI → AI-driven clinical decision making	0.114	0.091	1.252	0.210
**Total indirect effects**				
AI reduces workload → AI-driven clinical decision making	0.070	0.061	1.227	0.220
Clinical experience (control variable) → AI-driven clinical decision making	0.005	0.008	0.665	0.506
Perception of AI risk → AI-driven clinical decision making	−0.008	0.012	0.555	0.579
**Specific indirect effects**				
Clinical experience (control variable) → Trust in AI → AI-driven clinical decision making	0.005	0.008	0.665	0.506
Perception of AI risk → Trust in AI → AI-driven clinical decision making	−0.008	0.012	0.555	0.579
AI reduces workload → Trust in AI → AI-driven clinical decision making	0.070	0.061	1.227	0.220
**Total effects**				
AI reduces workload → AI-driven clinical decision making	0.739	0.069	10.688	<0.001
AI reduces workload → Trust in AI	0.660	0.080	8.252	<0.001
Clinical experience (control variable) → AI-driven clinical decision making	0.031	0.046	0.703	0.482
Clinical experience (control variable) → Trust in AI	0.048	0.056	0.888	0.375
Perception of AI risk → AI-driven clinical decision making	−0.347	0.067	5.287	<0.001
Perception of AI risk → Trust in AI	−0.062	0.070	0.854	0.393
Trust in AI → AI-driven clinical decision making	0.109	0.091	1.252	0.210

## Data Availability

The anonymized data from this study can be obtained upon request from the corresponding author. However, the data are not accessible to the public due to privacy and confidentiality concerns regarding participant information.
